# Prediction of protein-protein interactions from amino acid sequences using a novel multi-scale continuous and discontinuous feature set

**DOI:** 10.1186/1471-2105-15-S15-S9

**Published:** 2014-12-03

**Authors:** Zhu-Hong You, Lin Zhu, Chun-Hou Zheng, Hong-Jie Yu, Su-Ping Deng, Zhen Ji

**Affiliations:** 1 College of Computer Science and Software Engineering, Shenzhen University, Shenzhen, Guangdong 518060, China; 2 Department of Computer Science and Technology, Tongji University, Shanghai 201804, China; 3 College of Electrical Engineering and Automation, Anhui University, Hefei, Anhui 230601, China; 4 Department of Mathematics, School of Science, Anhui Science and Technology University, Fengyang, Anhui 233100, China

## Abstract

**Background:**

Identifying protein-protein interactions (PPIs) is essential for elucidating protein functions and understanding the molecular mechanisms inside the cell. However, the experimental methods for detecting PPIs are both time-consuming and expensive. Therefore, computational prediction of protein interactions are becoming increasingly popular, which can provide an inexpensive way of predicting the most likely set of interactions at the entire proteome scale, and can be used to complement experimental approaches. Although much progress has already been achieved in this direction, the problem is still far from being solved and new approaches are still required to overcome the limitations of the current prediction models.

**Results:**

In this work, a sequence-based approach is developed by combining a novel Multi-scale Continuous and Discontinuous (MCD) feature representation and Support Vector Machine (SVM). The MCD representation gives adequate consideration to the interactions between sequentially distant but spatially close amino acid residues, thus it can sufficiently capture multiple overlapping continuous and discontinuous binding patterns within a protein sequence. An effective feature selection method mRMR was employed to construct an optimized and more discriminative feature set by excluding redundant features. Finally, a prediction model is trained and tested based on SVM algorithm to predict the interaction probability of protein pairs.

**Conclusions:**

When performed on the yeast PPIs data set, the proposed approach achieved 91.36% prediction accuracy with 91.94% precision at the sensitivity of 90.67%. Extensive experiments are conducted to compare our method with the existing sequence-based method. Experimental results show that the performance of our predictor is better than several other state-of-the-art predictors, whose average prediction accuracy is 84.91%, sensitivity is 83.24%, and precision is 86.12%. Achieved results show that the proposed approach is very promising for predicting PPI, so it can be a useful supplementary tool for future proteomics studies. The source code and the datasets are freely available at http://csse.szu.edu.cn/staff/youzh/MCDPPI.zip for academic use.

## Background

Protein-protein interactions (PPIs) play key roles in various cellular processes, including metabolic cycles, DNA transcription and replication, and signalling cascades. Correctly identifying and characterizing protein interactions are critical for understanding the molecular mechanisms inside the cell [1]. In recent years, the researchers developed a couple of innovative techniques for identifying the interactions among proteins [1-3]. Due to the progress in high-throughput biological techniques such as Mass Spectrometric (MS), Tandem Affinity Purification (TAP) [1,4,3] and other large-scale experimental approaches for PPIs identification, an immense amount of PPIs data for different organisms has been accumulated [1-5].

However, the high-throughput experimental approaches are time consuming and expensive. Thus, current PPIs data generated with experimental methods only cover a small fraction of the complete PPI networks [6]. In addition, high-throughput biological techniques suffer from high false negative and false positive rates [6-10]. Therefore it is very important and urgent to develop the efficient and reliable computational approaches to facilitate the detection of protein interactions [11-13].

A number of computational techniques have been developed to provide either complementary information or supporting evidence to experimental methods [14-17]. Existing approaches typically use binary classification frameworks that differ in the features used to represent protein pairs. Different protein attributes or feature sources, such as protein domains, gene neighbourhood, phylogenetic profiles, gene expression, and literature mining knowledge are employed to infer protein interactions[6-8,11,18-25]. There are also approaches that integrate the interaction information from a couple of different biological data sources[26]. However, if the pre-knowledge about the proteins is not available the aforementioned approaches cannot be implemented.

Recently, a number of attempts which derive information directly from amino acid sequence have been made to develop computational model to help in discovering new PPIs [7,8,11,13,27-30]. The experimental results in previous works showed that the information of protein primary sequences alone is sufficient to detect protein interactions [7,11,13]. Shen et al. developed an automatic and excellent identification system for predicting PPIs based on protein amino acids sequence information [13]. In their study, the twenty protein amino acids were firstly clustered into seven clustering according to their dipoles and volumes of the side chains, and then we use the conjoint triad feature to represent of the given protein sequence based on the classification of amino acids. This approach achieves a high prediction accuracy of 83.9% on human PPIs data set. However, Shen's work cannot takes neighbouring effect into account and it is generally agreed that the interactions among proteins occur in the discontinuous amino acids segments of the protein sequence. Lately, Guo et al. employed auto covariance (AC) transformation method to consider the discontinuous amino acids segments in the sequence [11]. When applied to predict *saccharomyces cerevisiae *PPIs, their approach achieved a high prediction accuracy of 86.55%. In our previous studies, our methods which used autocorrelation descriptors and correlation coefficient also yielded good prediction performance [8,31,32].

In this study, a novel feature representation method for prediction of PPIs is proposed. We hypothesize that the continuous and discontinuous amino acids segments play an important role in determining the interactions between proteins. For example, discontinuous regions consist of amino acid residues remote from each other in primary protein sequence, yet spatially proximate in protein three-dimensional structure, which determines the interaction of proteins. In other words, the proposed protein representation method account for the interactions between sequentially distant but spatially close amino acid residues, thus it be able to adequately capture multiple overlapping continuous and discontinuous binding patterns within protein sequence.

To sum up, in this paper we propose a sequence-based approach for the prediction of protein-protein interactions using support vector machine (SVM) combined with a novel multi-scale continuous and discontinuous protein feature representation. In order to reduce the dimensionality of data and improve the accuracy of the predictor, an effective feature selection method minimum redundancy maximum relevance (mRMR) is employed to select a compact and discriminative new feature subset [33]. The *Saccharomyces cerevisiae *PPI dataset was employed to evaluate the performance of the proposed method. The experiment results demonstrate that our approach yielded 91.36% prediction accuracy with 91.94% precision at the sensitivity of90.67%. Our proposed method was also evaluated using the independent dataset of the *Helicobacter pylori *PPIs and achieved a high overall accuracy of 84.91%, which further demonstrates the effectiveness of the proposed method.

## Results

In this section, we first briefly introduce the PPIs datasets which is employed to evaluate the proposed method. Then we discuss the evaluation strategies used in performance comparisons. Finally, we analyse the experimental results and compare our results with the related research.

### Benchmark PPI datasets

To evaluate the performance of the proposed method, the PPIs dataset collected from yeast core subset of Database of Interacting Proteins (DIP) has been employed. This dataset is originally derived by Guo *et al*. and consists of 11,188 protein pairs, where half are from the positive dataset and half are from the negative dataset [11]. It should be noticed that the protein pairs which contain a protein with fewer than fifty residues or have ≥40% sequence identity were removed in our PPIs dataset; the remaining 5,594 protein pairs comprise the final positive dataset. Choosing negative examples is a very important for training a predictor of PPIs. The common method is based on annotations of cellular localization. In this study, the 5,594 protein pairs occurring in two different subcellular localizations were chosen as negative PPIs dataset.

### Evaluation measures

In the experiment, the five-fold cross-validation was employed to evaluate the prediction performance of the proposed method. More specifically, the PPIs dataset is randomly divided into five equally sized subsets, and then each subset is used as a testing set in turn, while the other four subsets are used for training set.

Four evaluation metrics, sensitivity (SN), precision (PE), overall accuracy (ACC) and Matthews Correlation Coefficient (MCC) are used to measure the prediction performance of our method. They are defined as follows:

(1) $$ACC=\frac{TP+TN}{TP+FP+TN+FN}$$

(2) $$SN=\frac{TP}{TP+FN}$$

(3) $$PE=\frac{TP}{TP+FP}$$

(4) $$MCC=\frac{TP\times TN-FP\times FN}{\sqrt{\left(TP+FN\right)\times \left(TN+FP\right)\times \left(TP+FP\right)\times \left(TN+FN\right)}}$$

where TP, FP, TN and FN refer to number of true positive, number of false positive, number of true negative and number of false negative PPIs, respectively. MCC is considered to be the most robust metric of any class prediction method. An MCC equal to 0 is regarded as a completely random prediction, whereas 1 is regarded as a perfect prediction.

### Experimental setting

In this paper, the proposed sequence-based PPI predictor was implemented using MATLAB platform. For SVM algorithm, the implementation of LIBSVM available from http://www.csie.ntu.edu.tw/~cjlin/libsvm was utilized, which was originally developed by Chang and Lin [34]. The Radial Basis Function was chosen as the kernel function and the optimized parameters (C,γ) were obtained with a grid search approach. Regarding mRMR, the implementation by Peng and Ding available from http://penglab.janelia.org/proj/mRMR/ was used. All the simulations were carried out on a computer with 3.1 GHz 2-core CPU, 6 GB memory and Windows operating system.

### Prediction performance of proposed model

The DIP PPIs data which investigated in Guo *et al*. was adopted to evaluate the performance of the proposed model [11]. Proper parameters setting can improve the SVM classification accuracy; therefore the corresponding parameters for SVM were firstly optimized. Here, two parameters, C and gamma (γ), were determined using the grid search approach within a limited range. To guarantee that the experimental results are valid and can be generalized for making predictions regarding new data, the dataset is randomly partitioned into training and independent testing sets via a 5-fold cross validation. Each of the five subsets acts as an independent holdout testing dataset for the model trained with the rest of four subsets. Thus five models were generated for the five sets of data. The advantages of cross validation are that the impact of data dependency is minimized and the reliability of the results can be improved.

The prediction performance of SVM predictor with MCD representation of protein sequence across five runs are shown in Table 1, compared with several published results for the same dataset. From Table 1 we observed that high overall accuracy in the range of 91.01%-92.00% is obtained for the proposed model. To better evaluate the prediction performance of the proposed method, other four evaluation metrics including Sensitivity, Precision, MCC, and AUC are calculated in the study. It can be observed from Table 1 that the proposed method yielded good prediction performance with an average Sensitivity value of 90.67%, Precision value of 91.94%, ,AUC value of 97.07%and MCC value of 84.21%. In addition, we can observed from Table 1 that the standard deviation of overall accuracy, precision, sensitivity, MCC and AUC are as low as 0.36, 0.62, 0.69, 0.59 and 0.12, respectively.

**Table 1:** Comparison of the prediction performance by the proposed method and some state-of-the-art works on the yeast dataset.

*Model*	Test set	**SN***(%)*	**PE***(%)*	**ACC***(%)*	**MCC***(%)*	**AUC***(%)*
Our method	1	91.66	92.40	92.00	85.28	97.15
	2	90.49	92.01	91.11	83.79	96.91
	3	91.13	91.05	91.19	83.94	96.97
	4	90.41	91.48	91.01	83.64	97.07
	5	89.64	92.76	91.47	84.38	97.23
	Average	**90.67 ± 0.69**	**91.94 ± 0.62**	**91.36 ± 0.36**	**84.21 ± 0.59**	**97.07 ± 0.12**

Guos' work	ACC	89.93 ± 3.68	88.87 ± 6.16	89.33 ± 2.67	N/A	N/A
	AC	87.30 ± 4.68	87.82 ± 4.33	87.36 ± 1.38	N/A	N/A

Zhous' work	SVM+LD	87.37 ± 0.22	89.50 ± 0.60	88.56 ± 0.33	77.15 ± 0.68	95.07 ± 0.39

Yangs' work	Cod1	75.81 ± 1.20	74.75 ± 1.23	75.08 ± 1.13	N/A	N/A
	Cod2	76.77 ± 0.69	82.17 ± 1.35	80.04 ± 1.06	N/A	N/A
	Cod3	78.14 ± 0.90	81.86 ± 0.99	80.41 ± 0.47	N/A	N/A
	Cod4	81.03 ± 1.74	90.24 ± 1.34	86.15 ± 1.17	N/A	N/A

Here, N/A means not available.

We further compared our method with Guo et al.[11], Zhou et al.[35] and Yang et al.[36], where the SVM, SVM and KNN was performed with the conventional Auto Covariance, Local Descriptor, and Local Descriptor representation as the input feature vectors, respectively. From Table 1, we can see that the performance of all of these methods with different machine learning model and sequence based feature representation are lower than ours, which indicates that our improvements are resulted from adopting the proposed MCD descriptor to represent the protein sequences. In a word, we may safely draw the conclusion that the proposed method generally outperforms the previous approaches with higher discrimination power for detecting PPIs based the information of protein amino acids sequences. Therefore, we can see clearly that our model is a much more efficient method for predicting PPIs compared with existing approaches methods. Therefore, it makes us be more convinced that our new method will be a useful tool for protein interaction prediction community.

### Comparing the prediction performance with other methods

We performed the PPIs dataset for *Helicobacter pylori *to highlight the advantage of the proposed model. The *H. pylori *PPIs dataset is originally derived by Martin et al. and composed of 1,458 interacting pair and 1,458 non-interacting pairs for a total of 2,916 protein pairs, [37]. This dataset is adopted to performs a comparison of proposed method with several existing works including phylogenetic bootstrap[38], HKNN [39], ensemble of HKNN [40], signature products [37] and boosting [31]. The methods of phylogenetic bootstrap, signature products and HKNN are based on individual classifier system to infer PPIs, while the methods of HKNN and boosting belong to ensemble-based classifiers. The average prediction results of ten-fold cross-validation over six different approaches are given in Table 2. From Table 2, we can see that the average prediction performance, i.e. accuracy, sensitivity, precision and MCC achieved by proposed predictor, are 84.91%, 83.24%, 86.12% and 74.4%, respectively. It demonstrates that our method outperforms all other individual classifier-based methods such as Phylogenetic bootstrap. It can be also observed that the proposed method clearly yields a comparable performance with the other ensemble classifier systems (i.e. Boosting and ensemble of HKNN). All these results demonstrate that our model substantially improves the performance in the prediction of PPIs.

**Table 2:** Performance comparison of different methods on the H

Methods	SN (%)	PE (%)	ACC (%)	MCC (%)
Phylogenetic bootstrap	69.8	80.2	75.8	N/A
Ensemble of HKNN	86.7	85	**86.6**	N/A
Signature products	79.9	85.7	83.4	N/A
Boosting	80.37	81.69	79.52	70.64
HKNN	**86**	84	84	N/A
Proposed method	83.24	**86.12**	84.91	**74.40**

### Incremental Feature Selection (IFS) and optimal feature subset

The incremental feature selection (IFS) procedure was used to find an optimal subset from the mRMR feature list generated above [41]. Suppose the total number of the features is . We can obtain feature subsets which are initiated from a subset containing one feature and generated by adding them one by one from the mRMR feature list. Then SVM predictors were constructed with 5-fold cross-validation based on the feature subsets. Finally the IFS curve of MCC to the different feature subset was plotted. An optimal feature subset was obtained with which the corresponding predictor yields the best MCC. The detailed analysis of the experimental results in this section and the IFS curve are available at the website: http://csse.szu.edu.cn/staff/youzh/MCDPPI.zip.

## Conclusions

In this study, we have proposed an efficient technique for predicting protein interactions from protein primary sequences by combining a novel multi-scale continuous and discontinuous (MCD) feature representation with SVM model. The MCD representation takes into account the factors that the PPIs usually occur in discontinuous segments in the protein sequence, where distant amino acid residues are brought into spatial proximity by protein folding. A protein sequence was characterized by a number of regions using MCD representation, which is capable of capturing multiple overlapping continuous and discontinuous binding patterns within a protein sequence. In order to reduce the noise and irrelevant features which affect the protein prediction performance, the mRMR method was adopted for feature selection. Experimental results show that our method performed significantly well in predicting protein interactions. Achieved results demonstrate that the proposed approach is very promising for predicting PPI and can be a useful supplementary tool to traditional experimental method.

## Methods

In this section, we introduce the proposed MCD-SVM approach for predicting protein interactions from protein primary sequences. The proposed approach to predict the PPIs is consisted of three steps: (1) Represent protein sequences as a vector by using the proposed multi-scale continuous and discontinuous (MCD) feature representation; (2) Minimum redundancy maximum relevance (mRMR) is utilized to do the feature selection; (3) SVM predictor is used to perform the protein interaction prediction tasks.

### Feature vector extraction

To successfully use the machine learning methods to predict PPIs from protein sequences, one of the most important computational challenges is how to effectively represent a protein sequence by a fixed length feature vector in which the important information content of proteins is fully encoded. Although researchers have proposed various sequence-based methods to predict new PPIs, one flaw of them is that the interactions information cannot be drawn from both continuous and discontinuous amino acids segments at the same time. To overcome this problem, in this study we propose a novel Multi-scale Continuous and Discontinuous (MCD) sequence representation approach to transform the protein sequences into feature vectors by using binary coding scheme. A multi-scale decomposition technique is used to divide protein sequence into multiple sequence segments of varying length to describe both continuous and discontinuous regions. Here, the continuous sequence segments are composed of residues which are local in the polypeptide sequence, while discontinuous regions consist of residues from different parts of the sequence, brought into spatial proximity by the folding of the protein to its native structure.

**Figure 1:**
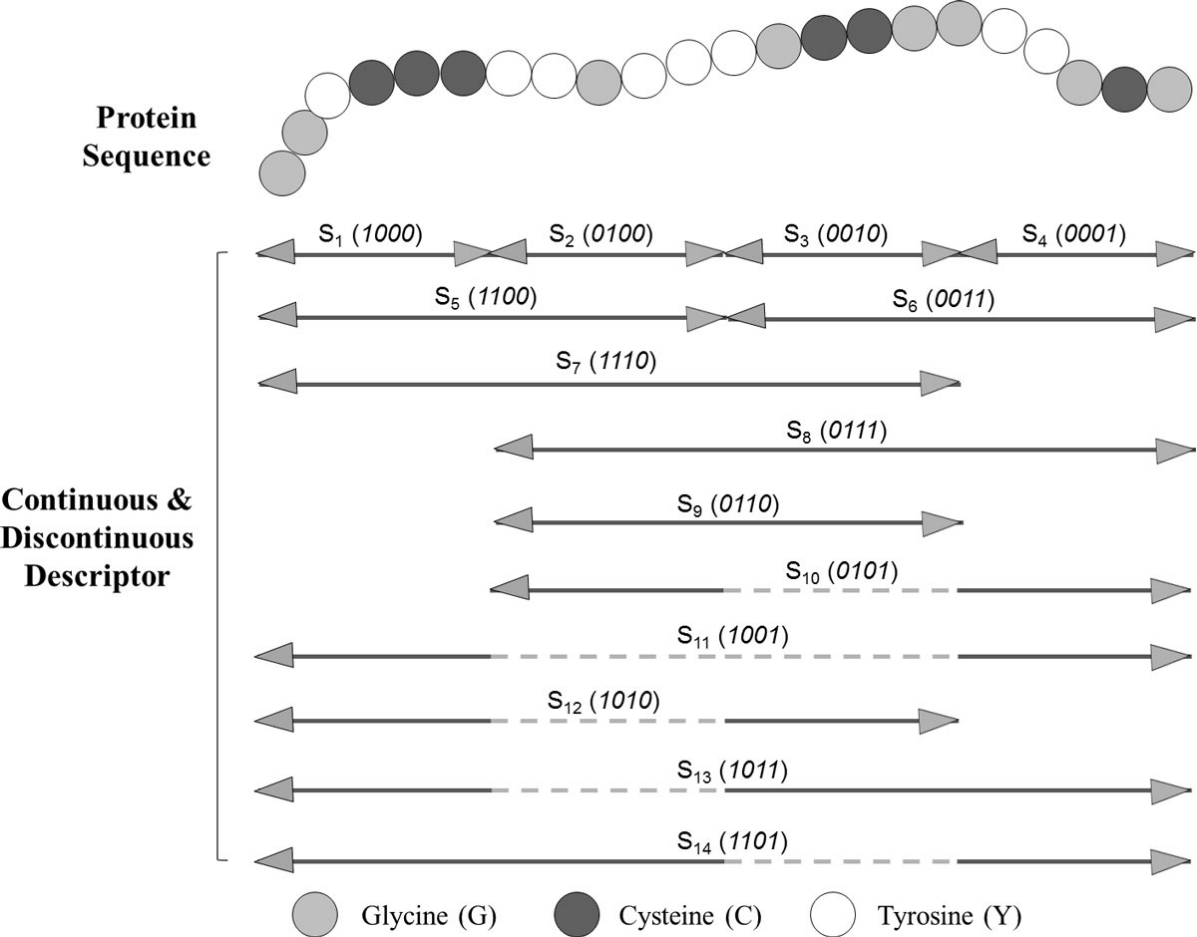
**The Schematic diagram for constructing continuous and discontinuous descriptor regions for a hypothetical protein sequence using 4-bit binary form**. Each protein sequence is divided into 14 (2^4-2) sub-sequences (regions S1 - S14) of varying length to represent multiple overlapping continuous or discontinuous segments.

In order to extract the interaction information, we first divided the entire protein sequence into a number of equal length segments. Then a novel binary coding scheme was adopted to construct a couple of continuous and discontinuous regions on the basis of above partition. For example, consider a protein sequence "GGYCCCYYGYYYGCCGGYYGCG" containing 22 residues. To represent the sequence by a feature vector, let us first divide each protein sequence into multiple regions. Refer to Figure 1, the protein sequence is divided into four equal length segments (denoted by S_1_, S_2_, S_3_and S_4_). Then it is encoded as a sequence of 1's and 0's of 4-bit binary form. In binary, these combinations are written as *0000, 0001, 0010, 0011, 0100, 0101, 0110, 0111, 1000, 1001, 1010, 1011, 1100, 1101, 1110, 1111*. The number of states of a group of bits can be found by the expression 2^n^, where *n *is the number of bits. It should be noticed that here 0 or 1 denote one of the four equal length region S_1 _- S_4 _is excluded or included in constructing the continuous or discontinuous regions respectively. For example, 0011 denotes a continuous region constructed by S_3 _and S_4 _(the final 50% of the sequence). Similarly, 1011 represents a discontinuous region constructed by S_1_, S_3 _and S_4 _(the first 25% and the final 50% of the sequence). These regions are illustrated in Figure 1.

It should be noticed that the proposed representation can be simply and conveniently edited at multiple scales, which offers a promising new approach for addressing these difficulties in a simple, unified, and theoretically sound way when present a protein sequence. For a given number of bits, each protein sequence may take on only a finite number of continuous or discontinuous regions. This limits the resolution of the sequence. If more bits are used for each protein sequence, then a higher degree of resolution is obtained. For example, if the protein sequence is encoded by 5-bit binary form, each protein sequence may take on 30 (2^5^-2) different regions. Higher bit encoding requires more storage for data and requires more computing resource to process.

For each continuous or discontinuous region, three types of descriptors, composition (C), transition (T) and distribution (D), are used to represent its characteristics. C is the number of amino acids of a particular property (e.g., hydrophobicity) divided by the total number of amino acids in a local region. T characterizes the percentage frequency with which amino acids of a particular property is followed by amino acids of another property. D measures the chain length within which the first, 25%, 50%, 75%, and 100% of the amino acids of a particular property are located, respectively [42].

The three descriptors can be calculated in the following ways. Firstly, in order to reduce the complexity inherent in the representation of the 20 standard amino acids, we firstly clustered them into seven groups based on the dipoles and volumes of the side chains. Amino acids within the same groups likely involve synonymous mutations because of their similar characteristics [13].The amino acids belonging to each group are shown in Table 3.

**Table 3:** Division of amino acids into seven groups based on the dipoles and volumes of the side chains.

Group 1	Group 2	Group 3	Group 4	Group 5	Group 6	Group 7
A,G,V	C	M,S,T,Y	F,I,L,P	H,N,Q,W	K,R	C

Then, every amino acid in each protein sequence is replaced by the index depending on its grouping. For example, protein sequence "GGYCCCYYGYYYGC-CGGYYGCG" is replaced by *1132223313331221133121 *based on this classification of amino acids. There are eight '1', six '2' and eight '3' in this protein sequence. The composition for these three symbols is 8 × 100%/(8+6+8) = 36.36%, 6 × 100%/(8+6+8) = 27.27% and 8 × 100%/(8+6+8) = 36.36%, respectively. There are 4 transitions from '1' to '2' or from '2' to '1' in this sequence, and the percentage frequency of these transitions is (4/21) ×100% = 19%. The transitions from '1' to '3' or from '3' to '1' in this sequence can similarly be calculated as (6/21) ×100% = 28.57%. The transitions from '2' to '3' or from '3' to '2' in this sequence can also similarly be calculated as (2/21) ×100% = 9.52%.

**Figure 2:**
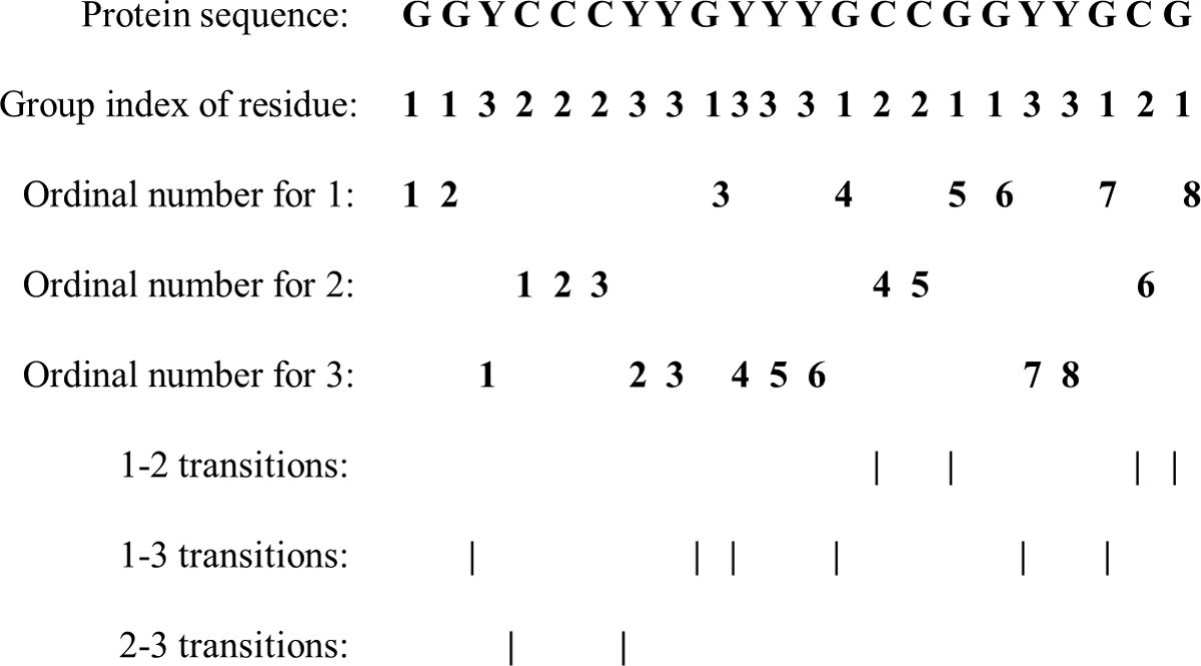
**Sequence of a hypothetic protein indicating the construction of composition, transition and distribution descriptors of a protein region**.

For distribution D, there are 8 residues encoded as "1" in the example of Figure 2, the positions for the first residue '1', the 2nd residue '1' (25% × 8 = 2), the 4th '1' residue (50% × 8 = 4), the 6th '1' (75% × 8 = 6) and the 8th residue '1' (100% × 8) in the encoded sequence are 1, 2, 13, 17, 22 respectively, so the D descriptors for '1' are: (1/22) ×100% = 4.55%, (2/22) ×100% = 9.09%, (13/22) ×100% = 59.09%, (17/22) ×100% = 77.27%, (22/22)×100% = 100%, respectively. Similarly, the D descriptor for '2' and '3' is (18.18%, 18.18%, 27.27%, 63.64%, 95.45%) and (13.64%, 31.82%, 45.45%, 54.55%, 86.36%), respectively.

For each continuous or discontinuous region, the three descriptors (C, T and D) were calculated and concatenated, and a total of 63 descriptors are generated: 7 for C, 21 ((7 × 6)/2) for T and 35 (7 × 5) for D. Then, all descriptors from 14 regions were concatenated and a total 882 dimensional vector has been built to represent each protein sequence. Finally, the protein pair is represented by concatenating the two vectors of two individual proteins. Thus, a 1764-dimentional vector has been constructed to character each protein pair and used as a feature vector for input into SVM classifier.

### Minimum redundancy maximum relevance (mRMR)

After the feature extraction procedure, all protein interaction and non-interaction pairs in benchmark datasets are converted into numerical feature vectors with the same dimension. In order to reduce feature abundance and computation complexity, the Minimum Redundancy Maximum Relevancy (mRMR) criterion was used in this study to select an optimal feature subset [33].

The mRMR was originally proposed by Peng *et al*. to deal with the microarray gene expression data processing [33]. It ranked features based on the trade-off between their relevance to the target concerned and the redundancy among the features themselves. Feature with the better trade-off between the maximum relevance to target and the minimum redundancy between features were considered as better features and would be selected in the final ordered list. The mRMR algorithm is described briefly below.

Mutual information (MI), which estimates how much a vector is related to another, was used to quantify both relevance and redundancy. The mutual information of two vectors  and, denoted as , could be calculated as below:

(5) $$I\left(x,y\right)=\iint p\left(x,y\right)\text{log}\frac{p\left(x,y\right)}{p\left(x\right)p\left(y\right)}$$

where p(x,y) is the joint probabilistic density function of  and .  and  are the margin probabilistic density function of  and, respectively.

Suppose $S_{F}$,  and $S_{T}$ denote the whole feature set containing all the features, the already selected feature set containing  features, and the to-be-selected feature set containing  features, respectively. The relevance  of the feature  in  with the target  can be calculated by

(6) D=I(f,c)

The redundancy  of the feature  in  with all the features in $S_{S}$ can be calculated by

(7) $$R=\frac{1}{m}\underset{f_{i}\in S_{S}}{\sum }I\left(f,f_{i}\right)$$

In order to let the feature $f_{j}$ in $S_{T}$ with the maximum relevance to target  and minimum redundancy among features, The mRMR feature selection framework attempts to optimize Equations (10) and (11) simultaneously through aggregating the two criterion functions into a single criterion function. MID (mutual information difference) or MIQ (mutual information quotient) criteria can be employed to solve the above optimization problem. In this study, Equations (6) and (7) are combined into the mRMR function:

(8) $$\underset{f_{j}\in S_{T}}{\text{max}}\left[I\left(f_{j},c\right)-\frac{1}{m}\underset{f_{i}\in S_{S}}{\sum }I\left(f_{j},f_{i}\right)\right]\;\left(j=1,2,...,n\right)$$

Given a dataset with  features, the mRMR feature evaluation will continue  rounds. Finally, an ordered feature set can be obtained in which each feature has a subscript index indicating at which round the feature is chosen. The earlier the feature has been selected in the evaluation, the smaller the index is and the better the feature is. The mRMR program could be downloaded from the website at http://penglab.janelia.org/proj/mRMR/.

### Support Vector Machine (SVM)

Support Vector Machine (SVM) is a classification and regression paradigm first developed by Vapnik [43]. It has attracted much research attention in these years due its demonstrated improved generalization performance over other techniques in many real world applications including bioinformatics [8]. The SVM originated from the idea of the structural risk minimization theory. The main difference between this technique and many other conventional classification techniques including neural networks is that it minimizes the structural risk instead of the empirical risk. The principle is based on the fact that minimizing an upper bound on the generalization error rather than minimizing the training error is expected to perform better. SVM training always seeks a global optimized solution and avoids over-fitting, so it has the ability to deal with a large number of features. A complete description to the theory of SVMs for pattern recognition is in Vapnik's book [43].

The basic idea of utilizing SVM model for classification can be stated briefly as follows. Firstly, map the original data  into a feature space  with high dimensionality through a linear or non-linear mapping function, which is relevant with the selection of the kernel function. Then, within the feature space from the first step, seek an optimized linear division, i.e. construct a hyper plane which separates the data into two classes.

Given a training dataset of instance-label pairs $\left\{x_{i},y_{i}\right\},i=1,2,...,N$ with input data $x_{i}\in R^{n}$ and labelled output data $y_{i}\in \left\{+1,-1\right\}$. The classification decision function implemented by SVM is represented in the following equation:

(9) $$y\left(x\right)=sign\left[\overset{N}{\underset{i=1}{\sum }}y_{i}\alpha_{i}\cdot K\left(x,x_{i}\right)+b\right]$$

where the coefficients $\alpha_{i}$ are obtained by solving the following convex Quadratic Programming (QP) problem:

(10) $$\text{Maximize}\;\overset{N}{\underset{i=1}{\sum }}\alpha_{i}-\frac{1}{2}\overset{N}{\underset{i=1}{\sum }}\overset{N}{\underset{j=1}{\sum }}\alpha_{i}\alpha_{j}\cdot y_{i}y_{j}\cdot K\left(x_{i},x_{j}\right)$$

(11) $$\text{Subject}\;\text{to}\;0\leq \alpha_{i}\leq C$$

(12) $$\overset{N}{\underset{i=1}{\sum }}\alpha_{i}y_{i}=0\;i=1,2,...,N.$$

In the equation (11),  is a regularization parameter which controls the tradeoff between margin and misclassification error. These $x_{j}$ are called Support Vectors only if the corresponding $\alpha_{j}>0$. In this work, Radial Basis Functions (RBF) kernel, $K\left(x_{i},x_{j}^{}\right)=\text{exp}\left(-\gamma {∥x_{i}-x_{j}^{}∥}^{2}\right)$, is applied, which has better boundary response and most high-dimensional data sets can be approximated by Gaussian like distributions. In the experiment we use the well-known software LIBSVM to classify the PPI dataset [34].

## Competing interests

The authors declare that they have no competing interests.

## Authors' contributions

ZY conceived the algorithm, carried out analyses, prepared the data sets, carried out experiments, and wrote the manuscript. LZ & CZ & ZM & BN & YL designed, performed and analysed experiments and wrote the manuscript. All authors read and approved the final manuscript.

## Declarations

The publication costs for this article were funded by the corresponding author's institution.

This article has been published as part of *BMC Bioinformatics *Volume 15 Supplement 15, 2014: Proceedings of the 2013 International Conference on Intelligent Computing (ICIC 2013). The full contents of the supplement are available online at http://www.biomedcentral.com/bmcbioinformatics/supplements/15/S15.
